# Robust Cost Volume Generation Method for Dense Stereo Matching in Endoscopic Scenarios

**DOI:** 10.3390/s23073427

**Published:** 2023-03-24

**Authors:** Yucheng Jiang, Zehua Dong, Songping Mai

**Affiliations:** 1 Shenzhen International Graduate School, Tsinghua University, Shenzhen 518055, China; jyc14@tsinghua.org.cn (Y.J.);; 2 Peng Cheng Laboratory, Shenzhen 518000, China

**Keywords:** binocular endoscope, cost volume generation, cross-scale propagation, radiometric invariant metrics, stereo matching

## Abstract

Stereo matching in binocular endoscopic scenarios is difficult due to the radiometric distortion caused by restricted light conditions. Traditional matching algorithms suffer from poor performance in challenging areas, while deep learning ones are limited by their generalizability and complexity. We introduce a non-deep learning cost volume generation method whose performance is close to a deep learning algorithm, but with far less computation. To deal with the radiometric distortion problem, the initial cost volume is constructed using two radiometric invariant cost metrics, the histogram of gradient angle and amplitude descriptors. Then we propose a new cross-scale propagation framework to improve the matching reliability in small homogenous regions without increasing the running time. The experimental results on the Middlebury Version 3 Benchmark show that the performance of the combination of our method and Local-Expansion, an optimization algorithm, ranks top among non-deep learning algorithms. Other quantitative experimental results on a surgical endoscopic dataset and our binocular endoscope show that the accuracy of the proposed algorithm is at the millimeter level which is comparable to the accuracy of deep learning algorithms. In addition, our method is 65 times faster than its deep learning counterpart in terms of cost volume generation.

## 1. Introduction

Stereo matching is to find the corresponding pixels along the horizontal epipolar lines in the rectified image pairs captured by the calibrated binocular cameras [1]. It is a low-level vision task because it is the basis for other 3D applications [2]. 3D reconstruction and measurement in confined space are considered to be the future directions of endoscopies that can be used in medical and industrial scenarios [3]. However, with the limits of light conditions, the matching results need to be robust to the radiometric distortions.

Many stereo methods have been proposed to get reliable matching results, so-called disparities, based on traditional or deep learning algorithms [4,5]. According to the current public benchmarks, deep learning algorithms are far ahead of traditional algorithms in terms of accuracy [6]. However, deep learning is very data-dependent, and difficult to guarantee the same good performance in real, non-dataset scenarios. Also, high computational complexity limits its real-time application in embedded devices [5]. On the other hand, although traditional algorithms have advantages in the above aspects, the reliability of the traditional matching algorithms needs to be improved, especially in challenging scenes, such as homogeneous [7] and radiometric distorted areas [8].

The cost volume serves to evaluate the pixel dissimilarities between a pixel in the base image and its corresponding pixel in the matching image [4]. Its width and height are equivalent to that of the image being matched, while the length of its third dimension is the maximum disparity. Both handcrafted and learning-based functions can be used to generate cost values in the volume, e.g., Local-Expansion [9] utilizing these two kinds of cost volumes [10] as its data term respectively. Previous research has demonstrated that the quality of matching performance is largely determined by the cost volume. However, generating a cost volume requires significant calculations. Thus, in this paper, we aim to produce a robust cost volume that is suitable for binocular endoscopic scenarios using traditional methods with reliability comparable to that of deep learning methods but with less computational complexity.

Traditional methods can be further divided into local and global ones based on different optimization goals [11]. Local methods focus on improving the cost volume’s reliability by getting support for the nearby pixels in the local region, while global methods iteratively minimize the energy function composed of the data term and the smoothness term. As the cost volume can be used as data terms with a few modifications, we name them to cost volume uniformly. Our algorithm framework consists of two parts: the proposed cost generation method generating cost volumes with high robustness and the cost optimization methods (local or global) optimizing the cost volumes to get accurate disparities.

Our cost generation method contains three consecutive processes. First, we build the initial cost volume using the histogram of gradient angles (HOG angle) and amplitudes (HOG amplitude) which are all radiometric invariant based on the linear radiation distortion model (Section 3.1) [12]. Then the noises of the cost volume are suppressed by the guided filter under the same linear assumption as HOG metrics (Section 3.2) [13]. The same underlying assumption ensures the effectiveness of filtering. The robustness of the cost volume in low-texture areas is further improved by the new cross-scale propagation scheme (Section 3.3). As for cost optimization methods, two traditional methods [9,14] are used to get the disparity results after the final cost volume generation, which proves its advantage in reliability (Section 3.4). Section 4 presents the quantitative experimental results on the commonly used Middlebury datasets, a newly proposed surgical endoscopic dataset and the real image pairs of standard objects captured by our binocular endoscope.

## 2. Related Works

Considering the aim of our method, we mainly review the literature related to cost volume generation in this section. Various cost metrics have been proposed to construct the initial cost volume. Absolute Intensity Differences (AD) and Squared Intensity Differences (SD) along with their summation forms are the early metrics with low computational complexities [15]. However, they suffer from low performance in the region with radiometric distortion. In comparison, Normalized Cross Correlation (NCC) is insensitive to contrast changes but causes edge-fatten issues [16]. The subsequent zero-mean form of these metrics shows higher robustness at the cost of increased computation [8]. Census Transform (CT), as a representative of non-parametric metrics, has a relatively simple calculation process but good accuracy [17]. Ref. [12] uses the histogram of oriented gradient (HOG) descriptor [18] as the cost metric for the first time and proves its radiometric invariant property under linear radiation distortion assumption. Furthermore, a random forest classifier is trained in [19] to improve the confidence of the combination of the conventional metrics.

Cost aggregation is a key step in the stereo matching framework that can be used to classify algorithms into three different categories: local [20,21], non-local [22,23], and semi-global [4]. Non-local and semi-global methods are similar as their aggregation processes are along particular paths. However, the former methods limit the aggregation process to areas of similar colors by adaptive aggregation weights, while the latter methods perform the aggregation along scan lines in the whole image. The aggregations in local methods are limited to fixed, multi-fixed, or adaptive windows around the center pixel based on the local consistency assumption, which can result in blurring in discontinuity regions [24]. Considering the same de-noising purpose, edge-preserving filters are introduced to improve the cost volume’s robustness, achieving impressive results. In this paper, we choose the guided filter with the small filter radius for three reasons: ensuring model consistency by using the same window size of the cost metric computation; preventing the error propagation during the filtering process in the large area; low computational complexity [25].

As for deep learning methods, some optimization algorithms [9,26] using the cost volume generated by MC-CNN [10] are proven to be able to get more accurate disparity results than those using traditional metrics. Ref. [27] calculates the cost volumes at multiple levels using the shared features extracted by CNN. To improve the models’ generalization abilities and save the running memory and runtime, cascade cost volume structures in a coarse-to-fine manner are proposed [28,29]. Furthermore, these models can be used in optical flow estimation tasks [30].

## 3. Materials and Methods

Our cost volume construction process consists of several steps similar to cost calculation and aggregation, the first two procedures in the classical local method [11]. Besides, we introduce the cross-scale propagation method to further improve the robustness of the cost volume.

### 3.1. Pixel-Wise Cost Calculation

Radiometric distortion is a common factor deteriorating the stereo matching accuracy, caused by the unavoidable different settings of the binocular cameras and the noises in the imaging process [8]. Although ZNCC (zero-mean normalized cross-correlation) and CT (Census Transform) have been verified to be robust cost metrics [31], we use the improved HOG cost considering the matching accuracy. The accuracy evaluation of these metrics will be discussed in the experimental section.

(1) $$g_{m}\left(p_{m}\right)=c\cdot g_{b}\left(p_{b}\right)+t$$

(2) $$\theta_{b}\left(p_{b}\right)=\arctan\left(Gy_{b}\left(p_{b}\right)/Gx_{b}\left(p_{b}\right)\right)$$

(3) $$\theta_{m}\left(p_{m}\right)=\arctan\left(Gy_{m}\left(p_{m}\right)/Gx_{m}\left(p_{m}\right)\right)$$

Based on the linear radiation distortion model (1) and the linearity assumption in the small window, $\theta_{b}\left(p_{b}\right)$ in (2) and $\theta_{m}\left(p_{m}\right)$ in (3), the angles of the gradient vectors, are radiometric invariant [12]. In (1), $g_{b}\left(p_{b}\right)$ and $g_{m}\left(p_{m}\right)$ are the gray intensities of the corresponding pixels $p_{b}$ in base images and $p_{m}$ in matching images. *c* and *t* are model coefficients. In (2) and (3), Gx and Gy are the horizontal and vertical gradients of the pixel *p*. Likewise, the subscripts are used to distinguish between the base and matching images.

**Figure 1:**
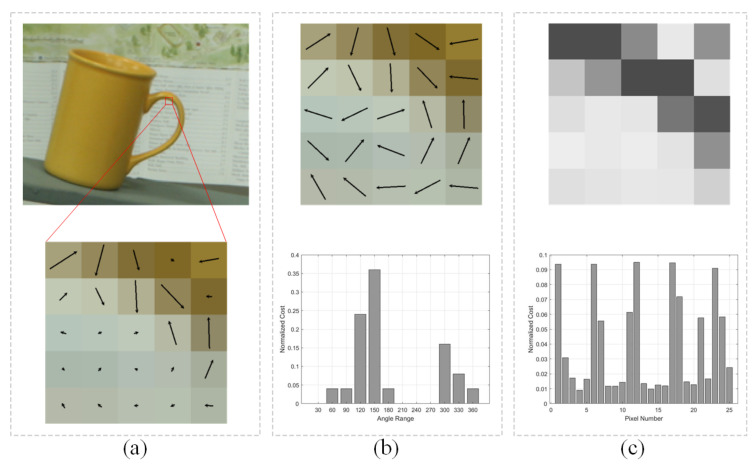
The HOG angle and relative HOG amplitude metrics calculation process. (**a**) The local window is centered at the pixel under calculation. The length and direction of each directed arrow represent the amplitude and angle of the gradient vector. (**b**) Construct the HOG angel by counting the number of vectors in each 30° bin. The value in each bin is normalized. (**c**) Construct the normalized HOG amplitude by dividing the amplitude of the gradient vector of each pixel by that of the center pixel. The value in each bin is also normalized.

As shown in Figure 1a,b, we first compute the gradient angles in the predefined window centered at the pixel *p*, then get the histogram of gradient angle by counting the number of vectors in each bin. The bins are obtained by dividing 360 degrees into 12 equal parts. After calculating each pixel’s normalized HOG angle in base and matching images, we can construct the cost volume $C_{HOG,angle}$ by,

(4) $$\begin{array}{c} C_{HOG,angle}\left(p_{b},d\right)=∥HOG_{angle}^{b}\left(p_{b}\right)-HOG_{angle}^{m}\left(p_{b}-d\right)∥ \end{array}$$

where $HOG_{angle}$ represents the angle histogram descriptor of the pixel *p* and *d* is the horizontal distance between corresponding pixels which is the disparity. The $∥*∥$ operator is the sum of the absolute difference of each bin in 12-dimensional vectors.

Unlike the CT metric used in [12], we further develop the relative HOG amplitude descriptors for compensating the important amplitude information of the gradient vector dismissed by the HOG angle descriptors. The relative amplitude ρ of the gradient vector is calculated by

(5) $$\rho_{b}=\frac{\sqrt{Gy_{b}{\left(p_{b}\right)}^{2}+Gx_{b}{\left(p_{b}\right)}^{2}}}{\sqrt{Gy_{b}{\left(p_{b,center}\right)}^{2}+Gx_{b}{\left(p_{b,center}\right)}^{2}}}$$

(6) $$\rho_{m}=\frac{\sqrt{Gy_{m}{\left(p_{m}\right)}^{2}+Gx_{m}{\left(p_{m}\right)}^{2}}}{\sqrt{Gy_{m}{\left(p_{m,center}\right)}^{2}+Gx_{m}{\left(p_{m,center}\right)}^{2}}}$$

where Gx and Gy have the same meaning as in (2) and (3). $p_{b,center}$ and $p_{m,center}$ represent the center pixels in the local windows in base and matching images respectively. We can get the amplitude histogram descriptor $HOG_{amplitude}$ by concatenating the relative amplitude value of each pixel in the window, as shown in Figure 1c. The gradient computation and the division operation relatively eliminate the influences of the coefficients *t* and *c* in the linear radiation distortion model, so $HOG_{amplitude}$ is also a radiometric invariant metric.

Then the corresponding cost volume is constructed by the following equation where the meanings of the symbols are the same as in (4).

(7) $$\begin{array}{c} C_{HOG,amplitude}\left(p_{b},d\right)=∥HOG_{amplitude}^{b}\left(p_{b}\right)-HOG_{amplitude}^{m}\left(p_{b}-d\right)∥ \end{array}$$

Finally, $C_{HOG,angle}\left(p_{b},d\right)$ and $C_{HOG,amplitude}\left(p_{b},d\right)$ are average fused to get the initial cost volume $C_{HOG}$.

**Figure 2:**
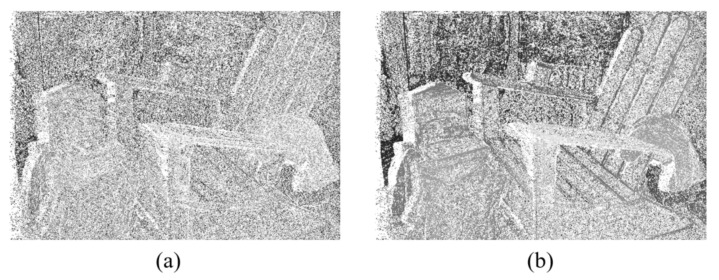
Disparity comparison of (**a**) generated from $C_{HOG,angle}$ and (**b**) generated from the fused cost volume of $C_{HOG,angle}$ and $C_{HOG,amplitude}$. White pixels represent the disparities failing the LRC check.

Figure 2 shows the difference in the disparities generated from $C_{HOG,angle}$ with and without the fusing of $C_{HOG,amplitude}$, using the WTA (winner-takes-all) strategy. The disparities that fail the LRC (left-right consistency) check are represented by white points [32]. Figure 2b shows significantly better performance than Figure 2a based on the LRC evaluation method.

**Figure 3:**
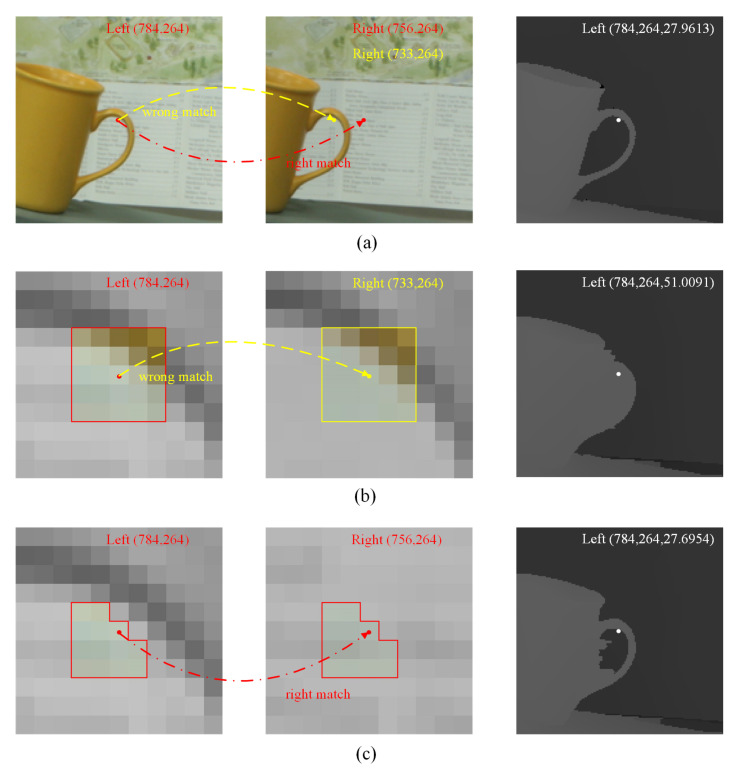
Illustration of the effectiveness of the adaptive window in the HOG descriptor building process. (**a**) The patches of the image pair Adirondack and the corresponding disparity ground truth in the Middlebury 2014 training dataset [6]. (**b**) The fixed window causes the wrong match of the specific pixel and the area of the cup handle. (**c**) The adaptive window reduces the matching errors in the same area.

The computational complexity of the HOG building process is relatively low when using the fixed window. However, both HOG angle and HOG amplitude descriptors are sensitive to the large distinguished values of the gradients. As illustrated in Figure 3a,b, the pixel near the cup handle in the left image tends to match the pixel in the same region of the right image, because of the top-right pixels’ dominant influences in the cost calculation process. The final disparity in Figure 3b shows that pixels in the background area inside the cup handle are given the wrong foreground disparity value.

To alleviate the influences of the distinguished gradients in the window, we set an RGB distance threshold and a gradient amplitude threshold. When building the HOG descriptors in the base image, if the color difference or the gradient difference between the local pixel and the center pixel exceeds the threshold, we adapt the shape of the local window to exclude this local position, and use this adaptive window to calculate the HOG metrics in the matching image. Figure 3c shows the result of this modification, the specific pixel matches the right position and the matching accuracy in the area of the cup handle is improved.

### 3.2. Cost Volume Filtering

The initial cost volume $C_{HOG}$ is usually full of noises which means the minimum position of the cost curve of a pixel may not be consistent with its correct disparity value. To improve the robustness of the cost volume, noise needs to be filtered by the cost volume filtering process. The joint bilateral filter [21] and the guided image filter [13] are the two commonly used edge-preserving filters. In the application of stereo matching, the guided image filter shows advantages in both the edge-preserving performance and the computational complexity [33]. More importantly, the guided filter is based on the same local liner model assumption as the HOG metric. So we use the following equation to compute the output of the guided filter [13].

(8) $$q_{i}=\frac{1}{\left|\omega \right|}\sum_{k:i\in \omega_{k}}\left(a_{k}I_{i}+b_{k}\right)$$

where $I_{i}$ is the value of the pixel *i* in the guided image and $q_{i}$ is the corresponding filter output. $a_{k}$ and $b_{k}$ are coefficients of the liner module of the window $\omega_{k}$, which are computed by the filter input and the guided image. $\left|\omega \right|$ is the number of pixels in the window $\omega_{k}$. And summation process involves all the windows containing the pixel $I_{i}$.

**Figure 4:**
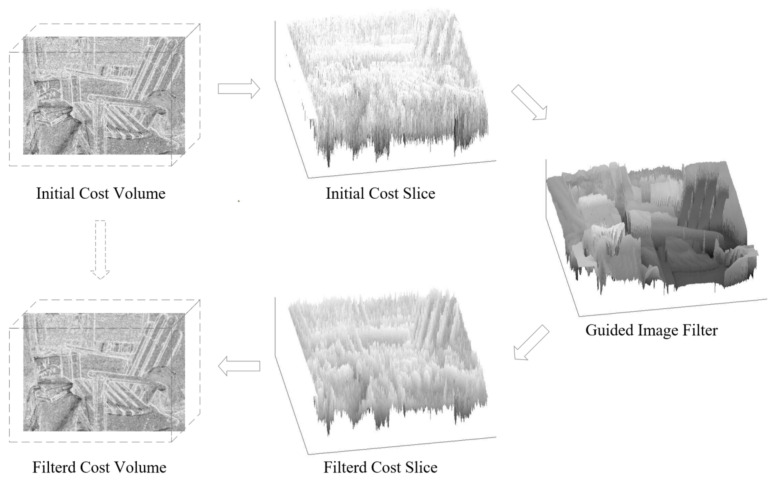
Cost Volume filtering process using the guided image filter.

The filtering process is shown in Figure 4. The initial cost volume is sliced along the disparity dimension. The size of each slice is the same as the matching image and the grayscale of the matching image is used as the guided image. The filtered cost slices are then combined as the filtered cost volume.

### 3.3. Cross-Scale Cost Volume Propagation

Matching the pixels in texture-less areas is difficult for stereo methods, especially for the local ones [34], because of the insufficient details to distinguish between similar pixels. Inspired by the fact that human eyes can use multi-scale information in the stereo matching process, a cross-scale cost aggregation framework is proposed in [35]. Although the aggregation framework’s effectiveness has been validated by experiments, it is not suitable for our method for two reasons: down-sampling in both width and height dimensions and fixed aggregation coefficients in all image areas.

Down-sampling in the horizontal direction of the input image will reduce the disparity range accordingly. The cost curves of the same pixel on coarse scales need to be interpolated to the same length as that on the finest scale. The robustness of the aggregated cost volume will decrease because of the errors introduced by the interpolations. So in our scheme, images are down-sampled in the vertical direction for the HOG descriptor to perceive more texture information, while the horizontal direction keeps unchanged considering the accuracy.

**Figure 5:**
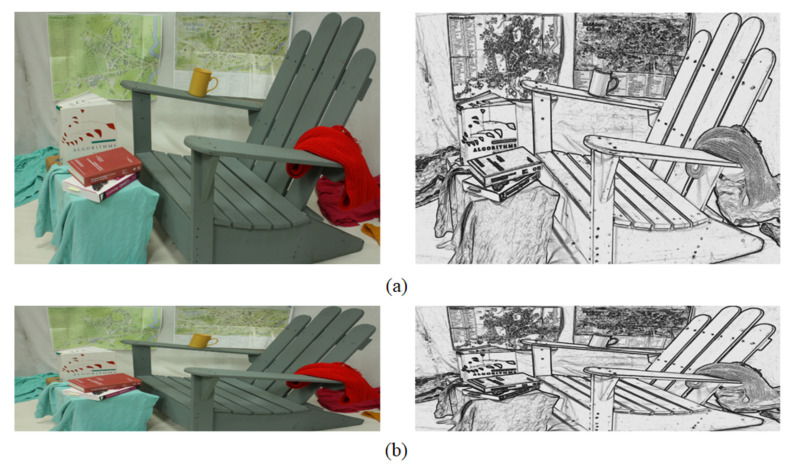
Homogeneity results of the Adirondack image on (**a**) full and (**b**) half scales.

Aggregation coefficients determine the proportion of the cost volume on each scale during the aggregation. The cost value may be more reliable in the complex texture areas of the image on the finer scale because of the loss of details by down-sampling. So we raise the proportion of the cost volume on the finer scale in the inhomogeneous areas. We adopt the method in [36] to determine the homogeneity of each area whose basic ideas are to convolve the pixels in the image with a Gaussian kernel sign-flipped in the horizontal or vertical direction and to use the sum of squares of the two directional convolution results as the indicator of homogeneity. The horizontal sign-flipped Gaussian kernel is calculated by

(9) $$f_{H}\left(x,y\right)=\left\{\begin{array}{c} \frac{1}{2\pi \sigma_{x}\sigma_{y}}\cdot e^{-(\frac{x^{2}}{2\sigma_{x}^{2}}+\frac{y^{2}}{2\sigma_{y}^{2}})},x\geq 0\\ -\frac{1}{2\pi \sigma_{x}\sigma_{y}}\cdot e^{-(\frac{x^{2}}{2\sigma_{x}^{2}}+\frac{y^{2}}{2\sigma_{y}^{2}})},x<0 \end{array}\right.$$

where *x* and *y* represent the pixel position in the convolution window. $\sigma_{x}$ and $\sigma_{y}$ control the shapes of the Gaussian kernel in two directions. As shown in Figure 5, the colors of complex textured areas are darker than those of simple texture areas.

**Figure 6:**
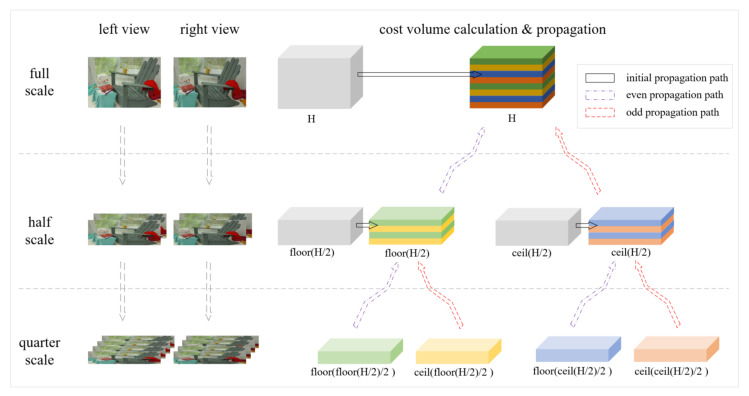
Illustration of the image down-sampling process and the cost volume propagation process.

We change the word aggregation to propagation as the name of the process because of the directional properties as shown in the right part of Figure 6. First, the input images are down-sampled from finest to coarsest scales. Then, after the initial cost volumes are generated on each scale, the propagation starts along the inverse direction of the down-sampling process. There are three propagation paths for each cost volume to be propagated:

1. From the initial cost volume of the same pixels on the same scale (black solid arrow in right part of Figure 6);
2. From the initial or propagated cost volume of the pixels on its even lines on the coarser scale (purple dot dash arrow);
3. From the initial or propagated cost volume of the pixels on its odd lines on the coarser scale (red dash arrow).

The cost volumes from path 2 and path 3 are propagated to the even and odd rows of the finer scale cost volume respectively and fused with the cost volume from path 1. The fusion weights are computed using (9). We can get the final cost volume on the finest scale after the whole propagation process is finished.

Although increasing the number of scales results in a proportional rise in computational complexity, the total running time remains relatively constant due to the ability to parallelize the calculation of the cost volume and its fusion weights at each scale.

### 3.4. Cost Volume Optimization

The above calculation process of the final cost volume is mainly based on the local linear assumption, so the sizes of the windows are limited to small enough to prevent error propagation from nonlinear areas. However, on the other hand, the lack of neighborhood information leads to relatively poor disparity results, so optimization methods are needed. Among the various existing optimization methods, we choose the AD-Census [14] in local methods and the Local-Expansion [9] in global methods to illustrate the effectiveness of the cost volume generated by our algorithm.

#### 3.4.1. AD-Census Optimization

The optimization procedure in the AD-Census method consists of two consecutive steps: cross-based cost aggregation and scanline optimization. The aggregation is based on the assumption that the pixels with similar colors have similar disparities in the local area, so the local information is taken into consideration. The scanline optimization uses the smoothness constraints that incorporate the global information.

**Figure 7:**
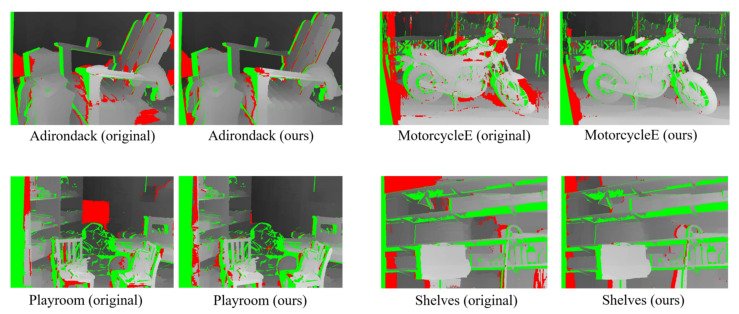
Comparisons of the disparities of the Middlebury 2014 dataset (quarter resolution) generated by the AD-Census optimization method using original cost volume (Left) and our final cost volume (Right).

We replaced the cost volume in [14], which was generated by summing the scaled AD and CT metrics, with our final cost volume. Figure 7 displays the results before and after the replacement, where green regions indicate occluded regions, and red regions represent pixels that failed the LRC check. It is evident that our cost volume is more robust in challenging datasets, such as MotorcycleE (which, according to the dataset website, is the exposure-changed version of Motorcycle).

#### 3.4.2. Local-Expansion Optimization

The combination of GC and spatial propagation in the Local-Expansion method minimizes the global energy function efficiently. Because the matching quality is greatly affected by the data term in the energy function, they replace the borrowed cost volumes in [9] with the ones generated by CNN [10] when testing on the Middlebury 2014 training dataset (interpolated to full precision). Here we use the propagated final cost volume as the input data term, and use the Local-Expansion optimization method to generate disparity results.

**Figure 8:**
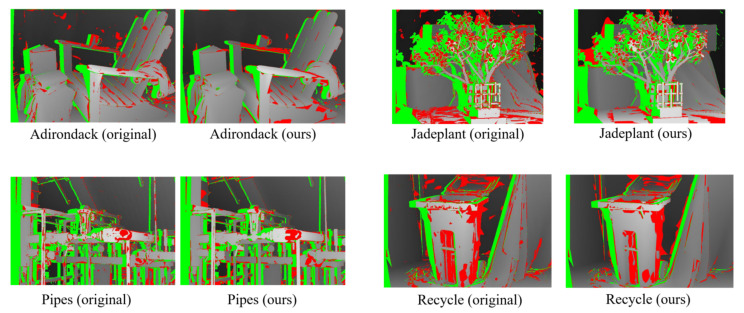
Comparisons of the disparities of the bad1.0 criteria generated by the Local-Expansion optimization method using CNN cost volume (Left) and our final cost volume (Right).

The disparity results are shown in Figure 8, where green regions indicate occluded areas, and red regions represent wrong matches (bad1.0) in non-occluded regions. Intuitively, the error rates of the results based on these two data terms are relatively close which shows the reliability of our cost volume. Further quantitative analysis is performed in the experimental section.

## 4. Results and Discussion

Our method is designed to generate robust cost volumes using the improved handcrafted functions, HOG. In this section, we use the Local-Expansion algorithm to optimize the generated cost volumes and to get final disparity results. The combination of these two methods is designed to estimate the depth information from the images captured by the binocular endoscope which is the practical application scenario of our algorithm. Considering the similarities between the endoscopic scenes and the indoor scenes in terms of low and repetitive textures, we mainly use the Middlebury 2014 dataset to evaluate the accuracy of our algorithm quantitatively.

We set the fusion ratio of the two kinds of HOG metrics to 1:1 in the initial cost volume generation process. The window sizes of histogram statistics and guided filter areas are both set to 5×5 to ensure the consistency of the liner model. We use three scales in the cost volume propagation step considering the performance and the amount of computation. The parameters of the Gaussian kernel used in homogeneity determination and the propagation weights of the simple and complex texture regions are set as follows: $\sigma_{x}=2.5$, $\sigma_{y}=1.5$, $\omega_{s}=0.3$, $\omega_{c}=0.7$. As for the parameters in Local-Expansion, the optimization method in our framework, we keep them consistent with the original paper [9].

### 4.1. Robustness Evaluation of Cost Metrics

**Figure 9:**
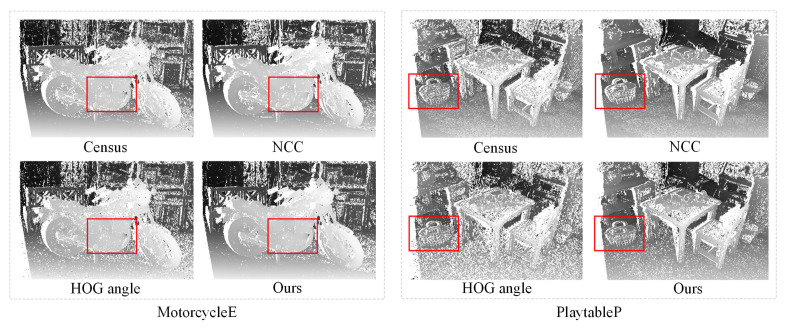
Comparison of the disparities generated by different cost metrics.

The initial cost volume which is directly calculated from the conventional cost metrics is the basis of further optimizations. To verify the performance advantage of the proposed HOG metric over other metrics, we compare the disparities generated by the proposed metric and other handcrafted radiometric invariant metrics using the WTA strategy without optimizations. Figure 9 illustrates the comparison results, where white points denote pixels that failed the LRC check. According to the proportion and distribution of white points in Figure 9, the proposed HOG metric shows greater reliability than the previous HOG angle metric (based on the ratio of the LRC passing pixels) and has better resolution in small texture-less areas (framed by red rectangles) compared with Census and NCC metrics.

Table 1 lists the quantitative comparison results generated by the ground truth disparities and the analyzing tool provided by the Middlebury dataset, where the numbers in bold are the results with the best performance in the listed cost metrics. These results show that our proposed metric outperforms other metrics in both accuracy (avgErr) and reliability (totBad).

### 4.2. Effectiveness Evaluation of Cross-Scale Propagation

**Figure 10:**
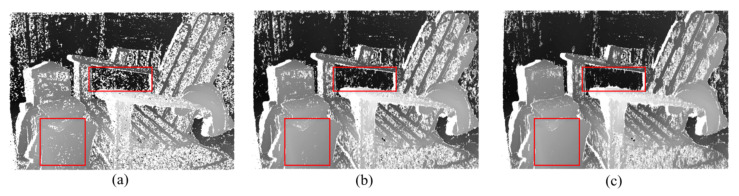
Comparison of the disparities generated by the propagation using (**a**) one scale, (**b**) two scales, and (**c**) three scales.

Cross-scale propagation is designed for improving the matching reliability in small homogeneous areas. Multi-scale images generated by down-sampling the original images enable the larger perception region along the vertical direction of the HOG descriptor and improve its resolving ability in the horizontal direction. Figure 10 displays the disparity comparisons after propagation using various numbers of scales, with white points indicating pixels that failed the LRC check. The ratio of bad matching pixels decreases as the number of scales increases, particularly in the region inside the red rectangles.

But too small scales will violate the local liner assumption and cause a large amount of calculation, so we set the number of scales to three for the balance. To be noted, the horizontal stripes in the disparity map caused by the fusion of its even and odd sub-scale cost volumes will be eliminated by the aggregation process in local methods or the smooth constraint in global methods in the optimization step.

### 4.3. Accuracy Evaluation on Middlebury Datasets

The ultimate purpose of the stereo matching methods is to get the high accuracy disparities, so we evaluate the accuracy of the disparities generated by the combined method (our cost volume generation method and Local-Expansion optimization) using the Middlebury Version 3 Benchmark.

As listed in Table 2, the results of our method are compared with SGM (semi-global method), INTS (non-local method), and original Local-Expansion (using the cost volumes generated by MC-CNN). Our method shows significant performance improvement on nearly all datasets compared with the first two pure traditional methods. As for original Local-Expansion, we achieve near or even better performance on most image pairs, but the performance drops terribly in specific scenes (italics in Table 2).

**Figure 11:**
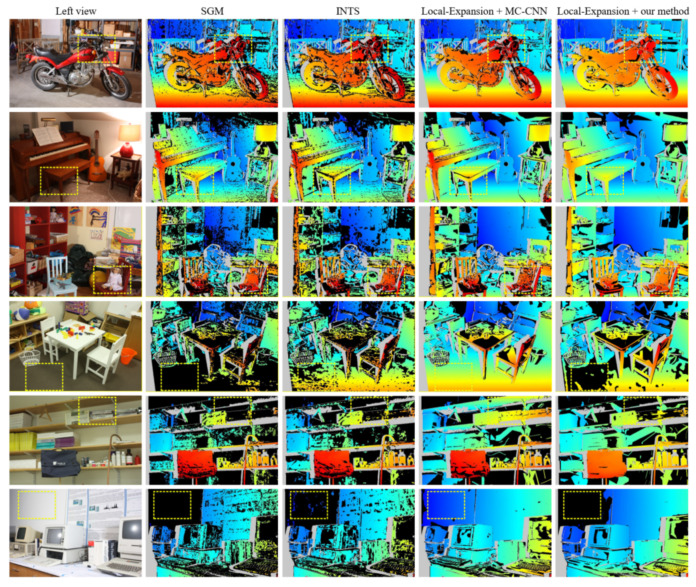
Comparison of disparities generated by SGM, INTS, origin Local-Expansion, and our method.

Figure 11 illustrates a comparison of the disparities generated by different algorithms, with the areas inside the yellow rectangles highlighting relatively noticeable performance differences. The top three rows correspond to the datasets MotorcycleE, Piano, and Playroom, where our method performs the best. In contrast, the bottom three rows correspond to the italicized entries in Table 2, indicating a drop in the performance of our method. We further analyze the reason for the performance changes of our proposed method. As shown in the top three rows of Figure 11, our method performs better than Local-Expansion in the textured and small texture-less areas (highlighted with yellow rectangles). However, in the large texture-less areas in the bottom three rows of Figure 11, our method performs far worse than Local-Expansion, even no better than SGM and INTS in the areas in the yellow rectangles. It is reasonable because our HOG metric cannot perceive a large area with the limitation of window sizes. Cross-scale propagation only improves the reliability of the costs in small texture-less areas. Based on our analysis, this shortcoming will not greatly affect the application of our method in the construction of endoscopic scenes because there will not be such large texture-less areas like floors and walls in these scenarios, especially in medical endoscopy. On the other hand, our method ranks top among the purely traditional algorithms in Middlebury Benchmark which proves its effectiveness.

To be noted, the ranks of our method drop significantly based on the bad2.0 and avgErr criteria, which means the percentage of “bad” pixels whose error is between 1.0 and 2.0 compared with the ground truth is relatively low. This proves the high reliability of our algorithm from another perspective because after removing the pixels with big matching errors using the LRC check, the accuracy of the remaining pixels will be high.

### 4.4. Accuracy Evaluation on Surgical Endoscopic Dataset

**Figure 12:**
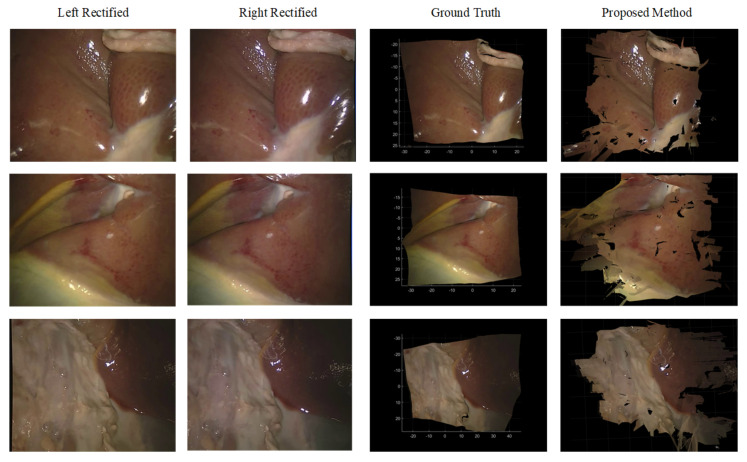
3D reconstruction results of SERV-CT datasets.

To evaluate the 3D reconstruction accuracy in real endoscopic scenario, we test the proposed combined method using the stereoscopic surgical endoscopic datasets SERV-CT [37]. With manual alignments of CT and endoscopic images, SERV-CT provides accurate depth results for 32 image pairs which can be used to evaluate stereo matching algorithm’s reconstruction accuracy. Figure 12 shows the comparison of 3D point clouds generated from disparities calculated using our combined method and ground truths. Because there are no large homogeneous regions in the endoscopic scenario, most of the non-occluded areas can be reconstructed well.

Based on the point clouds, we further calculate the 3D distance of the corresponding points and their average RMSE to represent the accuracy of the reconstruction. The quantitative comparison of accuracies of our method and various deep learning stereo matching algorithms are listed in Table 3. The reconstruction accuracy of our algorithm can reach about 5 mm, which exceeds that of some deep learning algorithms. This proves the effectiveness of our proposed method in this surgical endoscopic scenario.

### 4.5. Reconstruction Results Using Our Endoscope

**Figure 13:**
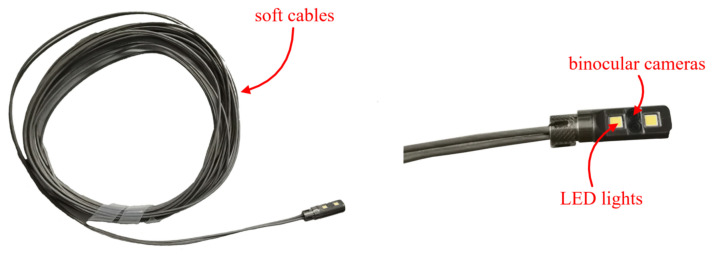
The real shot of the experimental binocular endoscope, see Supplementary Video.

The outer diameter and baseline length of our binocular endoscope are 6 mm and 2 mm. The resolution of a single image is 720×1280. As shown in Figure 13, because of the soft cable behind the cameras, it is ideal for 3D reconstruction and measurement in tight spaces, such as industrial and medical endoscopy.

**Figure 14:**
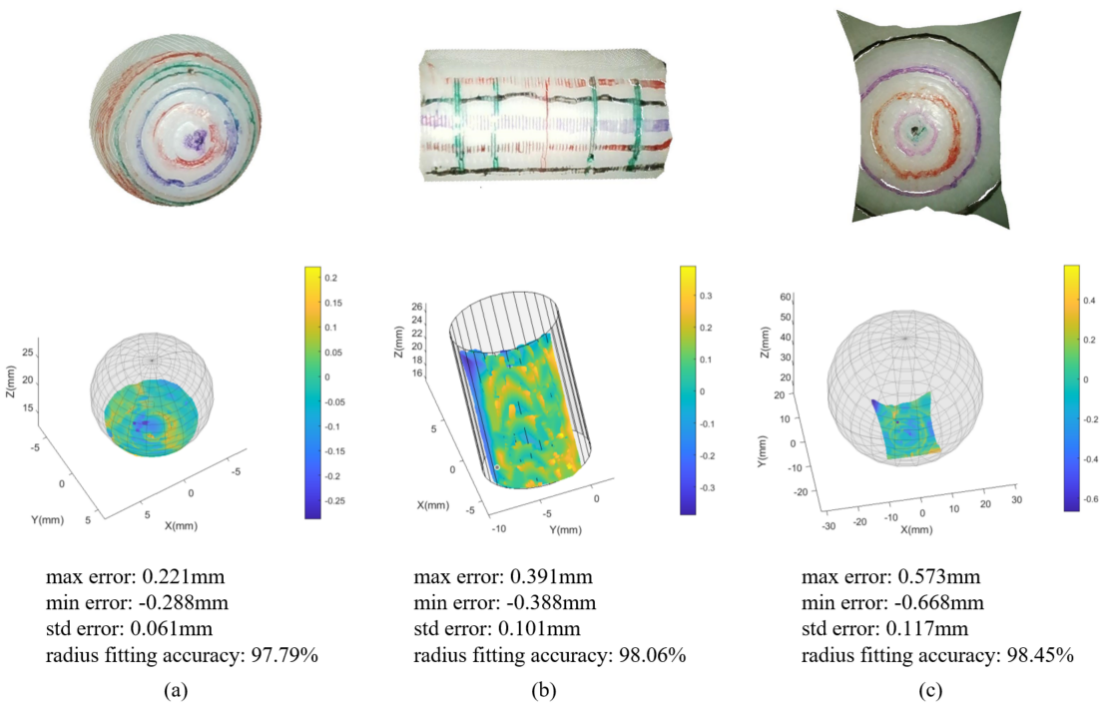
The point clouds and fitting results of (**a**) small sphere with a radius of 6 mm, (**b**) cylinder with a base radius of 5 mm, and (**c**) large sphere with a radius of 25 mm.

We perform surface fitting tests on two spheres and one cylinder that are 3D printed with the known parameters. The smaller size sphere (radius: 6 mm) and the cylinder (radius: 5 mm, height: 15 mm) are similar in size to the tumor, and the larger size sphere (radius: 25 mm) is used to approximate the surfaces inside the body. The point clouds and the fitting results of these three standard objects are shown in Figure 14 respectively. We paint some patterns on the surfaces of these objects to reduce the area of the homogeneous region. Such reconstruction accuracy (mm precision) enables the system to be used for measurement in endoscopic scenarios. Compared with the recent binocular endoscope reconstruction results [38], our system performs better in terms of the standard deviation errors of the fitting results.

### 4.6. Evaluation of Runtime

The runtime of the algorithm is another key factor affecting its application in practical scenarios. We port the MC-CNN algorithm provided by [9] and our method to the same GPU platform (NVIDIA GeForce RTX 2080 Ti) and compare their runtimes on cost volume generations using the Middlebury dataset. As presented in Table 4, the average runtime of MC-CNN is 65 times greater than that of our method, which clearly demonstrates the superiority of our approach in terms of computational efficiency.

## 5. Conclusions

In this paper, a novel non-deep learning cost generation algorithm is proposed to generate robust cost volumes for improving the matching accuracy in endoscopic scenarios. The new form of HOG metrics and the consecutive guided filter process guarantee the cost volume’s reliability in radiometric distorted regions, which shows the best performance among the radiometric invariant metrics. And the robustness of the cost volume in small homogeneous areas is improved by the proposed cross-scale propagation framework. The different propagation weights in textured and texture-less regions keep the accuracy of disparity details. Quantitative experimental results show the effectiveness of the algorithm combination of our method and the Local-Expansion algorithm on the datasets and real scenes. Combining the above advantages with the runtime saving by the relatively low computational complexity, this algorithm is suitable for applications in binocular endoscopic scenarios. However, our method cannot handle the large homogeneous areas well which will not be a problem in endoscopic applications, but it should be considered carefully in other scenarios. Also, the complexities of the optimization methods limit the overall efficiency, so the acceleration of these methods will be our future work.

## Figures and Tables

**Table 1:** Quantitate comparison between the conventional radiometric invariant metrics and the proposed HOG metric.

		bad1.0	Invalid	totBad	avgErr
MotorcycleE	Census	**10.82**	17.72	28.54	1.72
	NCC	12.91	16.88	29.79	1.67
	HOG ang	15.61	19.79	35.4	2.09
	Ours	12.82	**13.73**	**26.55**	**1.64**
PlaytableP	Census	13.69	27.72	41.41	2.98
	NCC	14.06	20.77	34.83	2.24
	HOG ang	19.24	35.15	54.39	4.25
	Ours	**13.42**	**20.22**	**33.64**	**2.19**

bad1.0: percentage of “bad” pixels whose error is greater than 1.0 compared with the ground truth; invalid: percentage of the pixels that failed the LRC check; totBad: the summation of the percentages of bad1.0 and invalid pixels; avgErr: average absolute error in pixels.

**Table 2:** Quantitative comparison between different stereo methods on Middlebury Version 3 datasets.

	${\mathrm{SGM}}_{92,98,108}$			${\mathrm{INTS}}_{82,89,78}$			$\mathrm{MCCNN},{\mathrm{LocalExp}}_{18,23,29}$			$\mathrm{OURS},{\mathrm{LocalExp}}_{31,69,75}$		
	bad1.0	bad2.0	Avgerr	bad1.0	bad2.0	Avgerr	bad1.0	bad2.0	Avgerr	bad1.0	bad2.0	Avgerr
Adirondack	29.1	15.3	2.06	24.5	11.0	1.17	5.90	1.20	0.55	7.23	3.78	0.79
ArtL	11.5	7.69	2.70	12.9	7.89	2.18	6.33	3.53	1.57	10.9	7.91	1.86
Jadeplant	28.1	18.1	9.17	28.5	18.6	7.15	19.0	8.95	4.8	19.09	9.8	6.29
Motorcycle	25.5	10.9	2.33	21.4	7.79	1.55	10.1	3.38	1.05	10.9	5.96	1.04
MotorcycleE	22.5	8.90	2.11	20.7	7.02	1.59	11.2	3.64	1.05	8.9	5.08	0.94
Piano	26.1	16.4	2.82	28.7	17.0	2.15	16.4	9.11	1.40	15.1	9.86	1.35
PianoL	42.1	29.1	5.70	41.0	27.1	5.29	25.8	14.7	2.46	29.6	24.8	6.02
Pipes	20.5	11.5	4.13	17.1	8.08	2.98	9.79	3.97	1.90	12.2	8.57	2.31
Playroom	38.3	21.7	3.11	38.4	20.9	2.61	22.0	9.07	1.75	19.4	12.3	1.95
*Playtable*	*67.4*	*52.5*	*25.6*	*40.7*	*25.2*	*4.35*	*16.4*	*6.45*	*1.16*	*38.2*	*28.9*	*4.71*
PlaytableP	24.7	15.8	2.60	25.9	15.4	2.03	12.2	5.85	1.00	13.9	9.46	1.53
Recycle	27.0	14.6	2.39	26.3	12.0	1.38	14.5	6.50	0.92	13.5	7.41	1.05
*Shelves*	*59.0*	*46.4*	*8.31*	*58.3*	*44.7*	*6.91*	*41.4*	*30.0*	*5.60*	*47.9*	*37.0*	*4.85*
Teddy	10.1	6.52	1.32	11.5	6.68	1.27	6.46	2.64	0.76	7.84	4.82	0.81
*Vintage*	*51.3*	*39.3*	*14.7*	*47.4*	*34.9*	*6.14*	*14.1*	*5.24*	*1.17*	*30.1*	*24.4*	*14.4*
Weight Avg	**28.2**	**15.3**	**4.83**	**26.4**	**15.0**	**2.89**	**13.7**	**6.52**	**1.69**	**16.2**	**10.9**	**2.71**

Our method performs much better than SGM and INTS on all datasets using bad1.0 criteria. Our method generates near or even better results on most image pairs using bad1.0 criteria, except the ones with large homogenous regions, shown in italics. The subscripts in the first row of the table represent the rankings of the algorithms in Middlebury Benchmark using three criteria respectively.

**Table 3:** Quantitative comparison of 3D reconstruction accuracy of various stereo matching algorithms.

	HSM (Level 1)	HSM (Level 2)	Hapnet	Stereo-UCL	Our Method
Mean RMSE Disparity (pixels)	**1.75** **(±0.53)**	2.13 (±0.69)	7.41 (±7.40)	9.24 (±2.96)	7.95 (±6.17)
Mean RMSE **3D Distance** (mm)	**3.18** **(±2.03)**	3.53 (±2.16)	6.01 (±4.07)	26.40 (±19.55)	3.50 (±2.70)

The data in bold is the best precision.

**Table 4:** Runtime comparison of the cost volume generation methods using Middlebury Version 3 Dataset.

Dataset	MC-CNN (s)	Ours (s)	Dataset	MC-CNN (s)	Ours (s)
Adirondack	28.292	0.415	Playroom	24.583	0.439
ArtL	4.697	0.109	Playtable	19.809	0.373
Jadeplant	-	0.811	PlaytableP	19.523	0.37
Motorcycle	31.191	0.402	Recycle	24.852	0.366
MotorcycleE	31.042	0.404	Shelves	26.948	0.353
Piano	21.863	0.356	Teddy	11.254	0.174
PianoL	23.011	0.357	Vintage	-	0.887
Pipes	30.983	0.439	Avg Time	22.927	0.351

The cost volume generation processes of Jadeplant and Vintage were failed using the MC-CNN method due to the limited resource of our GPU platform, so we excluded these two datasets when calculating the average runtime.

## Data Availability

Publicly available datasets were analyzed in this study. These data can be found here: vision.middlebury.edu (accessed on 11 April 2022) and https://www.ucl.ac.uk/interventional-surgical-sciences/weiss-open-research/weiss-open-data-server/serv-ct (accessed on 19 September 2022).

## Supplementary Materials

The following supporting information can be downloaded at: https://www.mdpi.com/article/10.3390/s23073427/s1.
